# A new standardization for the use of chicken embryo: selection of target from the phage display library and infection

**DOI:** 10.1007/s00253-024-13227-x

**Published:** 2024-07-10

**Authors:** Jessica Brito de Souza, Simone Sommerfeld, Hebréia Oliveira Almeida-Souza, Emília Rezende Vaz, Luciana Machado Bastos, Fabiana de Almeida Araújo Santos, Alessandra Castro Rodrigues, Alessandra Aparecida Medeiros-Ronchi, Luiz Ricardo Goulart, Belchiolina Beatriz Fonseca

**Affiliations:** 1 Postgraduate Program in Genetics and Biochemistry, Institute of Biotechnology, Federal University of Uberlândia, Uberlândia, Brazil; 2 Postgraduate Program in Veterinary Sciences, Faculty of Veterinary Medicine, Federal University of Uberlândia, Uberlândia, Brazil

**Keywords:** Filamentous phage M13, Bacterial infection, Animal model, Survivability

## Abstract

**Abstract:**

The filamentous bacteriophage M13KO7 (M13) is the most used in phage display (PD) technology and, like other phages, has been applied in several areas of medicine, agriculture, and in the food industry. One of the advantages is that they can modulate the immune response in the presence of pathogenic microorganisms, such as bacteria and viruses. This study evaluated the use of phage M13 in the chicken embryos model. We inoculated 13-day-old chicken embryos with *Salmonella* Pullorum (SP) and then evaluated survival for the presence of phage M13 or *E. coli* ER2738 (ECR) infected with M13. We found that the ECR bacterium inhibits SP multiplication in 0.32 (M13-infected ECR) or 0.44 log UFC/mL (M13-uninfected ECR) and that the ECR-free phage M13 from the PD library can be used in chicken embryo models. This work provides the use of the chicken embryo as a model to study systemic infection and can be employed as an analysis tool for various peptides that M13 can express from PD selection.

**Key points:**

• *SP-infected chicken embryo can be a helpful model of systemic infection for different tests.*

• *Phage M13 does not lead to embryonic mortality or cause serious injury to embryos.*

• *Phage M13 from the PD library can be used in chicken embryo model tests.*

## Introduction

Phage display (PD) technology consists of in vitro selection based on the presentation of peptides or proteins exposed on the surface of bacteriophages in the form of fusion proteins (Rahbarnia et al. 2017; Jiang et al. 2022). Bacteriophages are a type of virus that can carry out an infectious process in bacteria, fungi, actinomycetes, or spirochetes (Ge et al. 2020). PD applications are increasing and efficiently employed as phage therapy in veterinary medicine, agriculture, and food safety (Jamal et al. 2019).

Among the different types of bacteriophages used in PD, the most used is the filamentous phage M13KO7 (M13), which receives this name due to its filamentous appearance and dependence on pilus F in the infection process (Ebrahimizadeh and Rajabibazl 2014). Some of its applications are already well described in the literature, such as its use to evaluate antiviral activity (Nakakido et al. 2022) and stimulate the immune system by activating antigen-presenting cells (Dong et al. 2020). Furthermore, it proved that phages and peptides expressed and selected by the PD modulate the immune response against bacterial and viral infections (díaz-Valdés et al. 2011; Van Belleghem et al. 2019).

Given the importance of better understanding infectious processes and the search for the feasibility of experimental models, the chicken embryo (CE) is considered an accessible, inexpensive, and low-maintenance in vivo model. Moreover, it is easy to manipulate and allows a non-invasive follow-up during its development (Rashidi and Sottile 2009). Given all these advantages, this model has recently been used in several areas, such as evaluation of drug toxicity and distribution (Zosen et al. 2021; Ghimire et al. 2022), epigenetics (Bednarczyk et al. 2021), teratology (Wachholz et al. 2021), analysis of snake venom effects (Polláková et al. 2021), and bacterial infections (Li et al. 2019; Kosecka-Strojek et al. 2021).

The CE is a good model for tests with infection since it is possible to determine the pathogenicity of different bacteria (Gibbs et al. 2003; Oh et al. 2012; Blanco et al. 2018; Rezaee et al. 2021). Given the importance of using the PD to select ligands in several processes and the use of chicken embryos as a good study model to understand such mechanisms, this work aims to propose an experimental model for the utilization of phage M13 from the PD library in tests in an experimental model of chicken embryos. It would be helpful to have the chick embryo as an experimental model of pathogen-binding phages or other molecules for disease control.

## Materials and methods

This research was performed in the following laboratories of the Federal University of Uberlândia: Poultry Egg Incubation, Nanobiotechnology Luiz Ricardo Goulart Filho, Biochemistry, Laboratory of Infectious Diseases, and Animal Pathology. Project certified by the Ethics and Research with Animals Committee of the Federal University of Uberlândia (Nº 45/2022/CEUA/PROPP/REITO, process Nº23117.043271/2022–61).

### Evaluation of the ability of *E. coli* ER2738 and phage M13 to inhibit *S. * Pullorum in vitro

We developed a test to understand the *S. *Pulloruminfection in chicken embryos and then used this bacterium in the control group.

### Phage amplification and purification

In this work, we tested the *Escherichia coli* ER2738 (ECR), the bacteria that amplified phages from the phage display library, and phage M13, the wild-type phage without the peptide insert (also used in the phage display technology as a control). Amplification of wild phage M13 (New England Biolabs) was started by preparing a pre-inoculum containing one colony of ECR (New England Biolabs) at 37 °C in 50 mL of Luria Bertani (LB—Tryptone 10 g/L, yeast extract 5 g/L, NaCl 10 g/L) (Kasvi) culture medium with tetracycline (Sigma Chemical Co., 20 mg/mL) under stirring until reaching OD600 ~ 0.3. After, 10 µL of phage M13 was added and incubated at 37 °C overnight under shaking. Centrifuged the culture at 15,000 × g for 10 min and transferred the supernatant to a tube containing PEG/NaCl (20% polyethylene glycol 8000, Fluka, and 2.5 M NaCl Neon-sterile solution) and incubated at 4 °C overnight. Centrifuged the precipitate for 15 min at 15,000 × g, discarded the supernatant, and resuspended the pellet in PBS. Subsequently, it was centrifuged again for 10 min at 15,000 × g, then we transferred the supernatant to another tube containing PEG/NaCl, incubated for 1 h on ice and centrifuged 10 min at 15,000 × g. At last, resuspended the phage pellet with sterile PBS. After phage amplification, it was filtered on PES membrane with a pore size of 0.22 µm (K18-230, Kasvi) for further use during this work.

For bacterial inoculum, phage M13-infected and uninfected with ECR streaked on a plate containing LB enriched with IPTG (isopropyl β-d-1-thiogalactopyranoside-Ludwig Biotec) (0.5 mM) + X-gal (5-bromo4-chloro-3-indolyl β-d-galactopyranoside-Ludwig Biotec) (40 µg/mL) and tetracycline (Sigma Chemical Co., 20 mg/mL). After incubating for 24 h at 37 °C, 3 white colonies (not infected with the phage) were diluted in 10 mL of PBS and evaluated on the McFarland scale. In addition, inoculated 3 blue colonies (infected with the phage) into PBS. Both samples went through serial dilutions until it reaches inoculum amount. The exact amount was evaluated and confirmed by titrating the dilutions.

### Ability of ECR and M13 to inhibit *S. *Pullorum 

To propose an infection model, we used a SP isolated from free-range chickens by the Laboratory of Infectious Diseases at the Federal University of Uberlândia registered in the National System for the Management of Genetic Heritage and Associated Traditional Knowledge (SPU_SISGEN_28DD3D). The SP was cultured in nutrient agar (Kasvi) at 37 °C for 24 h. Before testing the embryos, we performed an in vitro test to evaluate the interaction between ECR and/or M13 incubated with SP.

To evaluate whether phage M13 can invade SP or influence its multiplication, incubated 500 µL of SP containing ~ 4.34 log CFU/mL with 500 µL of ECR or ECR infected with M13 (~ 4 log CFU/mL) at room temperature for ~ 20 min. Parallel, inoculated 1 mL of PBS containing 4.34 log CFU/mL of SP with 50 µL 10 log UFP/µL of phage M13 for ~ 20 min at room temperature. After this period, performed serial dilution and plated the samples on LB agar containing 0.5 mM IPTG, 40 µg/mL X-gal, and whether or not containing 20 mg/mL tetracycline. The medium with tetracycline inhibits the growth of SP but does not inhibit that of ECR. Performed SP colony count by the difference between the tetracycline-enriched and non-enriched plates.

### Evaluation of the inhibition ability of ECR and M13 on *S.* Pullorum in a chicken embryo model

#### Chicken embryos

The eggs line Hy-Line W36 were donated by New World Hatchery (Uberlândia, Brazil). Incubated the eggs in an artificial incubator (Premium Ecological®) at 37 °C, 58% humidity, and turned at a 2-h interval until 13 days of incubation (DI) when the tests started.

### Evaluation of the dose and age of SP inoculation in embryos

Since we know that SP leads to high mortality in embryos (Berhanu and Fulasa 2020), we did a pilot test to verify the best age and inoculation dose for them to suffer injury and for mortality to be equal to or lower than 60%. It is essential to evaluate the best age to work with the model. We used embryos at 13 and 14-day-old of incubation and before the experiment, 10 embryos were tested to the presence of SP by microbiology. The embryos were negative to SP and so, we inoculated with 6.13, 4.13, and 2.13 CFU/embryo via allantois (5 chicken embryo/group). The choice of age is because the embryo at this age already has an active immune system (Seto 1981) which facilitates understanding of the response to a challenge. The embryos were monitored daily for viability by ovoscopy. Four days after inoculation, euthanized embryos via cervical dislocation and evaluated macroscopic lesions.

### Evaluation of the effects of phage M13 and ECR on the embryo

To verify whether phage M13, phage M13-infected, and phage M13-uninfected ECR can be used on embryos without causing mortality, we performed a test on embryos. For this purpose, we inoculated the 13-day-old embryos via allantois with ~ 2.9 log CFU/embryo of M13-infected or uninfected ECR and 5 and 11 log CFU/embryo of purified phage M13. In parallel, a group of embryos received ~ 2.13 log CFU/embryo of SP, in addition to a negative control group. In each group, there were 5 embryos. The embryos were monitored daily by assessing viability by ovoscopy. After 4 days, euthanized the embryos via cervical dislocation and evaluated macroscopic lesions.

### Evaluation of the inhibitory capacity of phage M13 free or infecting ECR on SP infecting chicken embryos

To assess whether phage M13-free or infecting ECR interferes with mortality or injury caused by SP, embryos were infected with ~ 2.13 log CFU/embryo of SP via allantoic fluid at 13 days of incubation. After 1 h, we treated the embryos with ~ 2 log CFU/embryo of ECR, or ~ 2 log CFU/embryo of M13-infected ECR, or 11 log CFU/embryo of the M13 phage. We inserted SP-inoculated and negative control groups. Embryos were evaluated daily for viability by ovoscopy. At 17 days of incubation, we weighed the 21 surviving embryos, collected blood through the allantoic vessel, and performed macro- and microscopic analyses.

### Weight of the chicken embryos

Before the inoculation with SP, we numbered the eggs and recorded the weights. Then, at 17 DI, the CE were weighed immediately after collecting blood. As the embryo weight is related to the initial egg weight, we set the initial egg weight to 50 g, according to Ribeiro et al. (2020).

### Mortality and macroscopic evaluations

After the determined evaluation time, we checked and counted the embryos that died and determined the date of death according to the degree of development of the embryo. For the animals that were alive, we noted whether the annexes had the presence of circulatory changes, malformation, and/or color changes. We also performed an external evaluation on the embryos and evaluated the internal organs for circulatory changes, malformation, and color changes. We compared the treated groups with their respective control group.

### Histopathological changes

We performed a histopathological analysis of the liver and heart of all live embryos from the positive and negative groups in addition to 5 embryos from the SP-challenged and M13-infected ECR-treated group. The fragments of the liver and heart were fixed in 10% buffered formalin and processed for the preparation of histological slides stained with hematoxylin and eosin (HE) (Behmer and Tolosa 2003).

All slides from liver samples were analyzed by two experienced pathologists without knowledge of the treatment group. After lesions were identified and scored for severity, the slides for the control group were identified and re-evaluated for normality. The control samples were used as a guide for the normal histological appearance and the natural rate of lesion occurrence. All slides were re-examined, in comparison with a normal slide, to ensure accurate recognition and grading of lesions.

All liver slides were examined microscopically for histological evidence of degeneration, inflammation, and circulatory lesions (Molina et al. 2006). Severity scores were based on a scale of 0 to 3, which corresponded to normal, mild, moderate, and severe, respectively.

Hepatic lipidosis was scored as follows: 0, no detectable cytoplasmic vacuolation; 1, scattered individual vacuoles or low numbers of vacuoles within the cytoplasm of some hepatocytes; 2, clusters of vacuoles within the cytoplasm of many hepatocytes; 3, clearing of the cytoplasm because of advanced vacuolation in nearly all hepatocytes. The control samples were used as a guide for the normal histological appearance and the natural rate of lesion occurrence.

### ELISA

The levels of Interferon Gamma (IFN-γ), Interleukin-1 beta (IL-1β), and Interleukin 10 (IL-10) in the serum of chicken embryos were measured by enzyme-linked immunosorbent assay (ELISA) technique. High binding plates (Greiner Bio-One) were sensitized with embryo serum diluted (1:1) in 50 mM bicarbonate buffer (pH 8.6) for 1 h at 37 °C. After 3 washes with PBS-T (PBS + Tween 20 at 0.05%), the plates were blocked with 3% BSA in PBS for 1 h at 37 °C. Then, they were rewashed with PBS-T for 4 times. Then, we incubate the plates with the antibodies, rabbit anti-chicken IFN-γ IgG antibody (BioRad), rabbit anti-chicken IL-1β IgG antibody (BioRad) or IL-10 Polyclonal IgG antibody (Thermo), diluted (1:500) in 3% BSA + PBS for 1 h at 37 °C. After 4 washes with PBS-T, all plates were incubated with secondary goat anti-rabbit IgG HRP (Sigma) diluted (1:5000) in 3% BSA + PBS. Following this, washed 4 times with PBS-T, and the binding of the antibody/antigen was detected by adding 3,3′,5,5′-tetramethylbenzidine (TMB) substrate (Thermo Scientific). The reaction was stopped by the addition of 2 Normal (N) H_2_SO_4_. Reactivity was determined in a plate reader (Titertek Multiskan Plus, Flow Laboratories, USA) at a wavelength of 450 nm. During the reaction, we used different concentrations of recombinants IFN-γ, IL-1β, and IL-10 proteins (BD Biosciences, San Diego, CA) to construct the standard curve.

### Statistical analysis

We performed the Shapiro–Wilk test to evaluate if the data were parametric. The in vitro data were parametric, but the weight data were not. Therefore, we transformed the weight data using a square root transformation to follow a normal distribution and carried out the analyses. Then, we used ANOVA followed by the Tukey test. In mortality analysis, we perform the chi-square test, followed by the binomial between two proportions comparing all groups inoculated with SP. A relative standard curve was constructed from the absorbance values according to the control (recombinant protein IFN-γ, IL-1β, and IL-10). We interpolate the data using Pade (1,1) or hyperbolic approximant. After, the ANOVA test was followed by the Tukey test (*p* < 0.05) (GraphPad Prism 9.1).

## Results

### ECR can inhibit SP multiplication in vitro

The presence of the ECR bacterium, both alone and infected with phage M13, significantly decreased the amount of SP. In contrast, we did not observe the same result when only phage M13 was present (Table 1).

**Table 1:** Mean amount of SP (log CFU/mL) inoculated with ECR, ECR infected with M13, and M13

SP	SP (ECR)	SP (ECR + M13)	SP (M13)
4.34 (+ / − 0.08)a	3.9 (+ / − 0.12)b	4.02 (+ / − 0.06)b	4.37 (+ / − 0.13)a

SP, group infected with SP; SP (ECR), group of embryos challenged with *Salmonella* Pullorum (SP) inoculated with *Escherichia coli* ER2738 (ECR) not infected with phage M13; ECR+M13, group of embryos infected with SP and inoculated with ECR infected with phage M13; M13, group of embryos challenged with SP and inoculated with phage M13. Different letters show a statistical difference by ANOVA followed by Tukey’s test (*p* < 0.05)

### The dose of 2 log CFU/embryo of SP leads to a 60% mortality in 13- and 14-day-old embryos

**Fig. 1:**
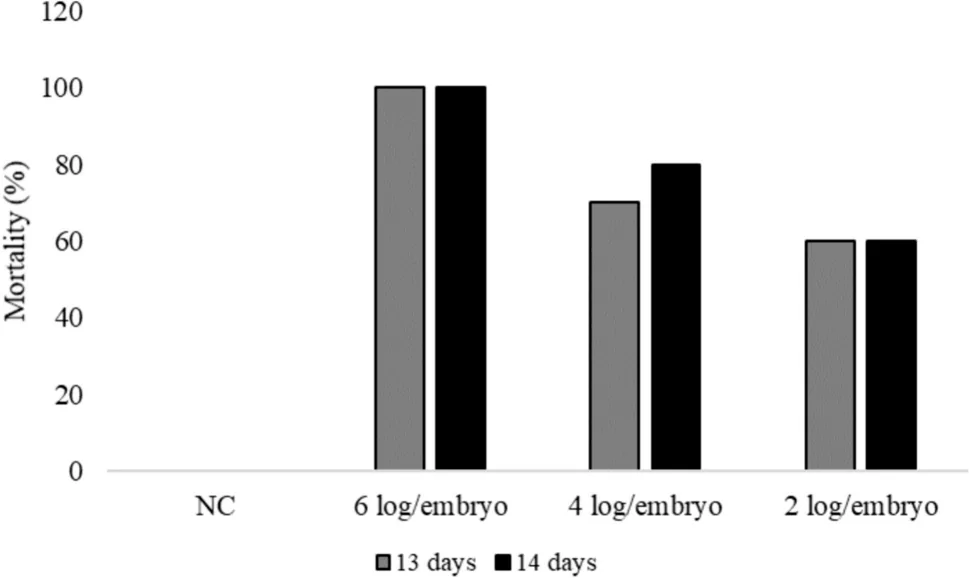
Percentage of embryos killed with different doses of SP at two different ages. NC, negative control group with PBS; 6 log/embryo, group inoculated with 6 log/embryo of SP; 4 log/embryo, group inoculated with 4 log/embryo of SP; 2 log/embryo, group inoculated with 2 log/embryo of SP

In the animal born, SP causes inflammation and a vertically transmitted disease that leads to lesions and mortality in old embryos. According to the pilot test performed, the embryos inoculated with SP at the lowest dose tested, 2 log CFU/embryo, showed the lowest mortality rate (Fig. 1). And after 4 days of inoculation, the embryos showed macroscopic lesions compared to the negative control, which suggests inflammation, among them thickening and increased redness of blood vessels, and an excess of excreta. Thus, we standardized on using 13-day-old embryos and 2 log CFU for the assays in this study.

### Free phage M13 or infecting ECR does not lead to mortality or serious lesions in embryos

From Table 2, phage M13 infecting ECR or free did not cause any death in the embryos. The only lesion found in the embryos treated with free phage or infecting ECR was excess uric acid in the embryos.

**Table 2:** Number of dead and injured embryos after inoculated with two doses of M13, M13-infected *E. coli* ER2738, and *E. coli* ER2738

	ECR + M13	ECR	M13 (5 PFU/µL)	M13 (11 PFU/ µL)	Nc	SP
24 h dead	0	0	0	0	0	1
Macroscopic alterations						Hemorrhagic embryo with membrane sticking
48 h dead	0	0	0	0	0	1
Macroscopic alterations						Hemorrhagic embryo with membrane sticking
Dead after 48 h	0	0	0	0	0	3
Macroscopic alterations	Excess uric acid in all	Excess uric acid in all	Excess uric acid in one embryo	Excess uric acid in all	All normal	Death 72 h after inoculation. Hemorrhagic and small
Total live	5	5	5	5	5	0

ECR + M13, group of embryos inoculated with ECR infected with phage M13; ECR, group of embryos inoculated with ECR not infected with phage M13; M13 (5 PFU/µL), group of embryos inoculated only with the phage at a concentration of 5 PFU/µL; M13 (11 PFU/µL), group of embryos inoculated only with the phage at the concentration of 11 PFU/µL; Nc, negative control (PBS); SP, control inoculated with SP

### ECR can decrease the mortality of embryos challenged with SP

According to Table 3, it was possible to observe that free phage M13 does not reduce the mortality of embryos inoculated with SP. In contrast, when the ECR becomes infected with phage M13, there is a significant reduction in the mortality rate in the face of infection caused by SP, which was present in all groups except the negative controls.

**Table 3:** Mortality rate (%) in embryos infected with SP and treated with free M13 and M13 infecting *E. coli*

NcPBS	NcM13	SP	SP (ECR)	SP (ECR + M13)	SP (M13)
0% (0/4)a	0% (0/4)a	75.0%(9/12)b	60.0%(3/5)b	25.0%(3/12)a	91.6% (11/12)b

% (x/y), mortality rate (number of dead/number of alive); NcPBS, negative control group with PBS; NcM13, negative control group with phage M13; SP, control group inoculated with SP; SP (ECR), group challenged with SP and inoculated with ECR not infected with phage M13 1 h later; SP (ECR+M13), group challenged with SP and inoculated with ECR infected with phage M13 1 h later; M13, group infected with SP and treated with phage M13 1 h later. Different letters show statistical difference between each group and SP group by chi-square test followed by binomial between two proportions (*p* < 0.05)

### ECR prevents weight loss of SP-treated embryos

**Fig. 2:**
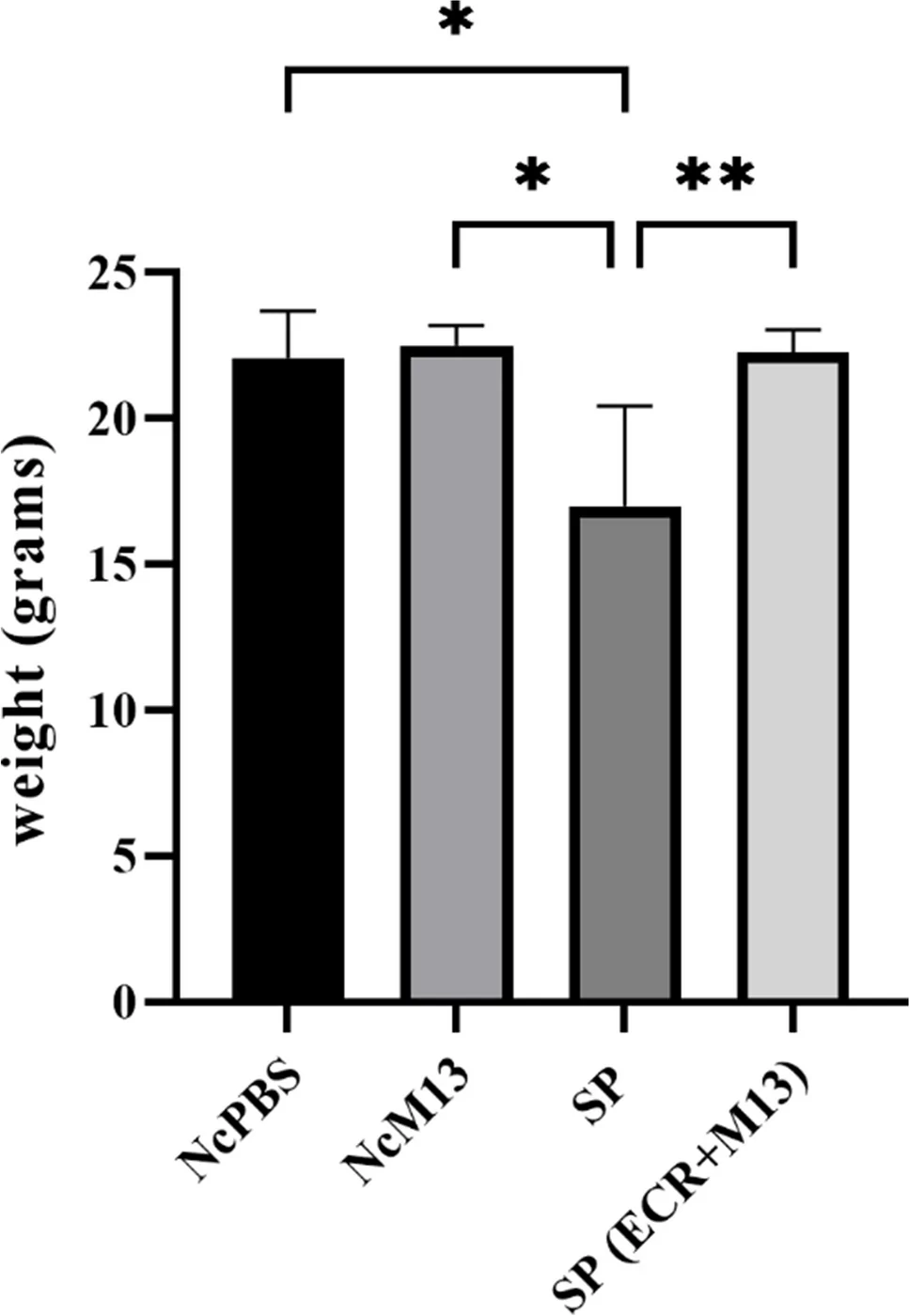
Weight of surviving embryos challenged with SP and treated or not with ECR infected with M13. NcPBS, negative control group with PBS; NcM13, negative control group with phage M13; SP, group inoculated with SP; SP (ECR + M13), infected by SP and treated with ECR infected with phage M13; M13, phage M13 only. Asterisk (*) shows a statistical difference (*p* < 0.05). The weights referring to group M13 and group ECR were not inserted in the graph because the embryos died, and the number of surviving embryos was not sufficient for statistical analysis

According to the graph shown in Fig. 2, it is possible to observe that the weight of embryos tends to decrease due to infection caused by SP. However, in the presence of ECR infected with phage M13, the embryos did not lose weight, and the values were close to those of the negative controls.

### Surviving embryos inoculated with SP and treated with ECR + M13 have no severe lesions

After 4 days of inoculation, we perform embryo diagnosis on the live embryos. In the group inoculated with SP, the 3 survivors showed excess excreta and thickening and greater redness of blood vessels, and 1 enlarged liver. In the group inoculated with SP and treated with the ECR bacteria infected with M13, only one embryo of the nine survivors had excess excreta. In the negative control group (embryo inoculated with M13), 2 of 4 embryos had excess excreta, while the negative control (inoculated with PBS) had no lesion.

### Histopathological changes

We found no histomorphometry changes in the negative control. Granulopoietic cells were present in all livers in connective tissues of hepatic portal spaces. Nevertheless, not among hepatoblasts and not all connective tissue areas in portal spaces were occupied by granulopoiesis foci. We observed mild lipidosis in all livers, even in the controls. In positive control, we found 1 of the 3 live embryos with hemorrhage and congestion in the liver. In group infected by SP and inoculated with M13-infected ECR, 1 of the 4 live embryos presented hemorrhage and congestion in the liver and heart, respectively. We performed only qualitative analysis.

### Embryos challenged with SP increase IL-10 secretion 4 days after inoculation, but when treated with ECR + M13, there is no increase in IL-10

**Fig. 3:**
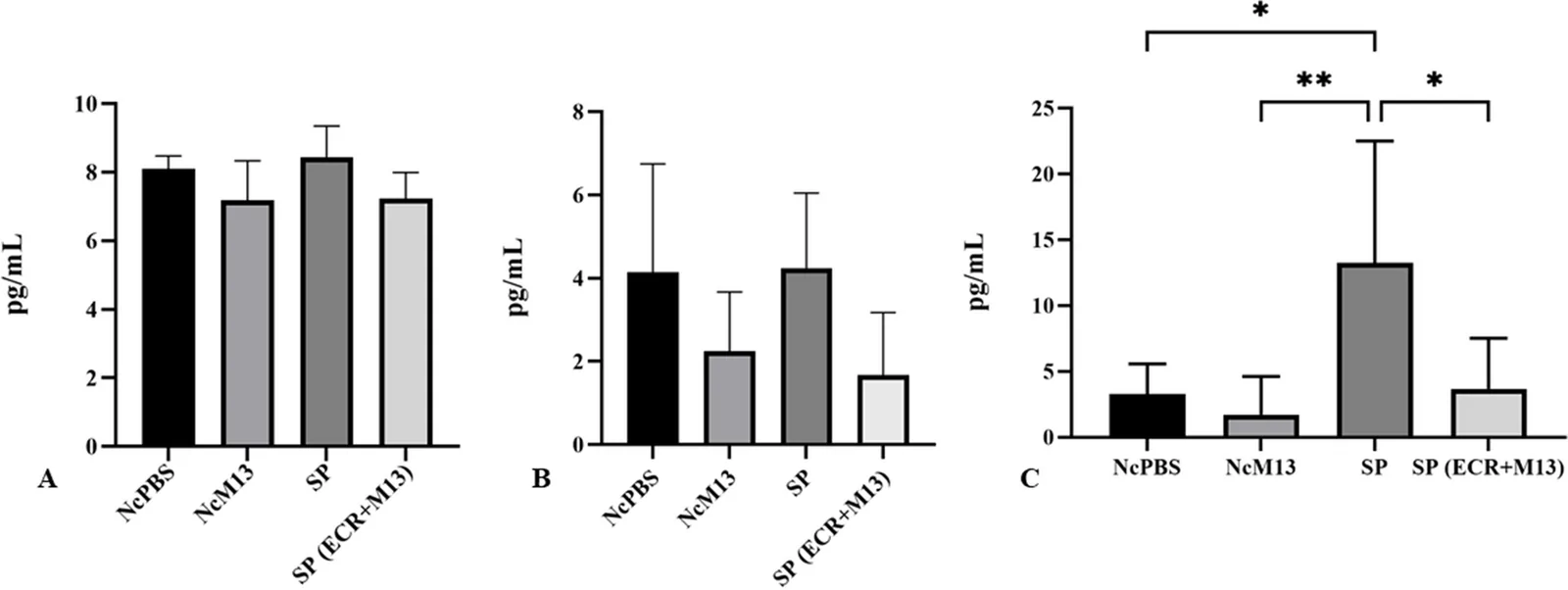
Dosage of inflammatory and anti-inflammatory cytokines in the serum of embryos. Levels (pg/mL) of IFN-γ (**A**), IL-1β (**B**), and IL-10 (**C**) in the serum of embryos. NcPBS, negative control group with PBS; NcM13, negative control group with phage M13; SP, group inoculated with SP; SP (ECR + M13), infected by SP and treated with ECR infected with phage M13; M13, phage M13 only. Asterisk (*) shows a statistical difference (*p* < 0.05). The cytokine levels referring to group M13 and group ECR were not inserted in the graph because the embryos died, and the number of surviving embryos was not sufficient for statistical analysis

There was no difference between IFN-γ and IL-1β levels and CP or CN (Fig. 3A and B). In contrast, the cytokine IL-10 had a significant decrease and a similar profile to the negative controls (Fig. 3C).

## Discussion

### In infection tests with SP, it is not interesting to use ECR infected with phage M13

After selecting phage ligands or mimetics by the PD library, the phages can be used as a screening test to choose the best targets by ELISA (da Silva et al. 2010). It can be very useful since peptide synthesis is still costly and time-consuming. There are still no studies of phages from the PD library that use screening tests for infection. It will have many applications because, besides the time and cost issues, the phage being a larger and more stable particle, could hit the target more successfully. To enable the use of phages, we have done an in vitro test inoculating the SP with the free phage or infecting ECR. We noticed a decrease in the amount of SP when inoculated with ECR (Table 1). This fact allows us to propose that in future works, if phages are used, they should be free of ECR so that the bacteria do not interfere with the tests. Although we only tested SP, it is possible that for other gram-negative bacteria, this event could also occur. The interaction between bacteria of different species happens through various mechanisms, such as competition for substrates and production of bacteriocins (Hawlena et al. 2012; Deng and Wang 2016). We cannot conclude what type of mechanism was used by ECR in the inhibition of SP, but other works show that probiotic *E. coli* strains can inhibit pathogenic bacteria (Setia et al. 2009; Fang et al. 2018; Hrala et al. 2021).

We also observed the in vitro results in tests on an embryonic model. When we challenged embryos with SP and then treated them with phage-infected ECR, it was possible to see a decrease in embryonic mortality. Embryos inoculated with SP showed a 75% mortality, while those inoculated with SP but treated with ECR had a 25% mortality (Table 3). This reduction was due to the presence of the bacteria since embryos challenged with SP and inoculated only with M13 showed no decrease in mortality. Lesions in embryos challenged with SP and treated with ECR infected with M13 were also mild. One embryo of the nine survivors showed increased excreta. However, 1 and 4 embryos in this group presented congestion and bleeding in liver and heart, respectively (Table 4). The weight of surviving embryos and the level of cytokine IL-10 of the SP-challenged group treated with ECR infected with M13 was similar to the negative control showing that the ECR was probably able to control the multiplication of SP in these embryos.

**Table 4:** Number of live chicken embryos with histomorphometric changes in liver and heart infected by SP or phage M13-infected ECR

Change	Organ	NcPBS	SP	SP (ECR + M13)
Degeneration	Liver	np	np	np
	Heart	0/4	0/3	0/5
Inflammation	Liver	0/4	0/3	0/5
	Heart	0/4	0/3	0/5
Bleeding	Liver	0/4	1/3	1/5
	Heart	0/4	0/3	4/5
Congestion	Liver	0/4	1/3	1/5
	Heart	0/4	0/3	4/5
Lipidosis	Liver	0/4	0/3	0/5
	Heart	np	np	np

^NcPBS, negative control group with PBS; SP, group inoculated with SP; SP (ECR+M13), inoculated with SP and treated with ECR infected with phage M13; np, not performed^

### Phage M13 does not lead to embryonic mortality or cause serious injury to embryos

Before we started testing, it was important to know if phage M13 caused damage or death in the embryos. Embryos inoculated with M13 at two doses (5 and 11PFU/embryo), ECR and ECR infected with M13 showed no mortality, and the only macroscopic change observed was excess uric acid (Table 2). Another experiment showed that phage M13 and ECR are harmless to hatchlings (de Almeida Araújo Santos et al. 2022). Our investigation found that tests with the phages can also be performed in chicken embryos as a potential infection model for evaluating PD-selected ligands.

### SP can be a model of infection in chicken embryos

SP is an avian-specific and vertically transmitted bacterium that causes severe injury to embryos, such as skin hemorrhage, subcutaneous edema, and increased mortality (Guo et al. 2017, 2019). Our intention was to find an embryonic period age and infective dose capable of not leading to the death of all embryos. We chose the ages of 13 and 14 days because before 11 days of incubation, there is death of 100% of embryos (data not shown) and because from that age on the embryos already have a more active immunity (Stefaniak et al. 2020). Our results show that embryos inoculated with the 6 and 4 log CFU doses of SP showed a higher mortality rate when compared to the groups inoculated with the dose of 2 log CFU/embryo (Fig. 1). At this dose, the mortality rate was similar, and thus, we decided to use the age of 13 days in the next phase to remove the biological material at 17 days of incubation. The intention was not to pass the age of 18 days of incubation because during this embryonic period, the embryo is already fully developed, becoming similar to an animal in experimental terms (Fonseca et al. 2021).

The decrease in weight of the SP-infected embryos (Fig. 2) and the lesions in the surviving embryos, such as hemorrhage and membrane sticking (Table 2), excess excreta, and hepatomegaly (described in Sect. 3.2.5), shows that these animals, although injured by the infection were able to survive trying to circumvent the inflammation caused by the bacteria. This result, together with the increase in the cytokine IL-10 in the surviving embryos (Fig. 3C), may be an attempt by the embryo’s immune system to modulate the inflammation caused by SP or the initiation of the Th2 type response similar to what occurs with the nascent animal (Tang et al. 2018; Foster et al. 2021). The inflammatory cytokines IFN-γ and IL-1β showed no increase (Fig. 3A and B). It probably happened because these cytokines are released at the onset of inflammation, characterizing the resistance phase of the disease. As the blood collection was 4 days after inoculation, already changed to the induction phase of SP modulation, other cytokines are participating in the process, such as IL-10 (Kogut and Arsenault 2017). This fact reinforces the idea that this cytokine can inhibit the production of inflammatory cytokines (Th1 type) during systemic dissemination to limit the inflammatory response (Rothwell et al. 2004; Tang et al. 2018).

From the histopathological analysis, we observed granulopoietic cells in all livers in connective tissues of hepatic portal spaces. Nevertheless, not among hepatoblasts and not all connective tissue areas in portal spaces were occupied by granulopoiesis foci. Despite the chicken fetal liver is not considered a relevant hematopoietic organ, as is the fetal liver in mammals (Wong and Cavey 1992, 1993), the presence of these granulopoietic foci was considered normal. Granulocytic differentiation in the connective tissue of portal spaces on the 15th day of incubation and onwards was reported by Guedes et al. (2014).

Even without showing inflammatory changes in the heart and liver, the chicks challenge with SP were smaller and depressed, with an increase in the vessels showing that it had a systemic inflammation that did not reach the tissues. In born animals, the histopathological lesions generated by SP are evident (Cheng et al. 2020), and the survival of the embryos in this study combined with the absence of liver and kidney damage (Table 4) together with the increase in serious IL-10 (Fig. 3) shows that embryos surviving the challenge with SP have a better response immune than those who died.

We observed mild lipidosis in all livers, even in the controls. Wong and Cavey (1992) reported that by 14th day of incubation, all hepatoblasts possess lipid and glycogen. The amount of fat in the hepatoblasts was considered at a normal level. The absence of any accompanying cytopathic effects in the liver allows the determination of their individual characteristics, not resulting from drug administration.

We also observed hepatic congestion and hemorrhage in some chicken embryos in the group treated with SP. This event is common in born animal (Shen et al. 2022). Our results indicate that SP is an interesting model of systemic infection in CE, and some embryo can be resistant to the disease progression.

### Phage M13 from the PD library can be used in chicken embryo model tests

The PD technology presents numerous advantages in the selection of ligands and structure mimetics of microorganisms, thus allowing both diagnosis and development of molecules for disease control (Sioud 2019). However, depending on the microorganism, the number of clones selected in the PD technology is high, and screening to choose the best ligands is essential. Although phage-ELISA can determine good ligands for diagnostic purposes (da Silva et al. 2010), this technique may not be interesting for understanding the ligand and host relationship, such as the infection and inflammation process. In this sense, cell culture is a useful tool, but considering the chicken embryo a more complex organism that allows the replication of numerous microorganisms such as viruses and bacteria (Farzaneh et al. 2017), this model has several advantages.

The advantages of chicken embryos over hatchlings are mainly related to cost, space, and some ease of handling (Garcia et al. 2021) and are currently accepted by the FDA in testing with some drugs (Kue et al. 2014). Based on the importance of the PD and the embryo as an experimental model, we propose a model of infection and suggest the embryo’s use in testing with the PD. Research with any system, whether organic or inorganic molecules, needs to be well standardized and to present guarantees of harmlessness so that the changes are well known. In this sense, this work clarifies that there are interferences of the ECR on the SP bacteria and that this may occur for other bacteria. Thus, our results show in vitro and in vivo models that in tests with infection, it is important that the M13 amplified in the ECR is purified. Another aspect that warrants the use of phage M13 from the purified PD library in tests with embryos is that M13 does not interfere with bacterial multiplication or the response generated by the embryo. This is seen when embryos inoculated with M13 alone did not lead to embryo mortality (Table 2) and when the mortality rate of the SP-inoculated and M13-treated embryo groups was equal to the SP-only inoculated group (Table 3). Unfortunately, the number of surviving embryos of the group challenged with SP and treated with M13 or ECR, although statistically similar to the group only challenged with SP, did not allow the analysis of the weight and level of cytokines produced as only one or two embryos survived, respectively. One can consider that the number of embryos for the ECR group was low, and a larger quantity is needed for better evaluation. However, the set of results allowed inferring that it is possible to use clones selected by the PD technology in embryo testing since M13 is innocuous and does not interfere with multiplication or bacterial action.

Chicken embryo may be a potential alternative for studying and selecting ligand-binding peptides from M13 phages selected from the PD library. The SP-infected chicken embryo can be a helpful model of systemic infection for different tests.

## Data Availability

All data will be available to the reviewer or editor upon request.
